# Mechanism of microbial action of the inoculated nitrogen-fixing bacterium for growth promotion and yield enhancement in rice (*Oryza sativa* L.)

**DOI:** 10.1007/s44307-024-00038-4

**Published:** 2024-09-19

**Authors:** Peng Li, Yunhe Tian, Kun Yang, Meijie Tian, Yi Zhu, Xinyu Chen, Ruiwen Hu, Tian Qin, Yongjun Liu, Shuguang Peng, Zhenxie Yi, Zhixuan Liu, Hejun Ao, Juan Li

**Affiliations:** 1 Hunan Soil and Fertilizer Institute, Hunan Academy of Agricultural Sciences, Changsha, 410125 China; 2 College of Agronomy, Hunan Agricultural University, Changsha, 410128 China; 3 Hunan Tobacco Science Institute, Changsha, 410004 China; 4 Hunan Tobacco Company Changde Branch, Changde, 415000 China; 5 Hunan Rice Research Institute, Hunan Academy of Agricultural Sciences, Changsha, 410125 China

**Keywords:** Rice yield, Nitrogen-fixing bacterium, *nifH* gene, Microbiome, α-diversity, Keystone taxa

## Abstract

The use of nitrogen-fixing bacteria in agriculture is increasingly recognized as a sustainable method to boost crop yields, reduce chemical fertilizer use, and improve soil health. However, the microbial mechanisms by which inoculation with nitrogen-fixing bacteria enhance rice production remain unclear. In this study, rice seedlings were inoculated with the nitrogen-fixing bacterium R3 (*Herbaspirillum*) at the rhizosphere during the seedling stage in a pot experiment using paddy soil. We investigated the effects of such inoculation on nutrient content in the rhizosphere soil, plant growth, and the nitrogen-fixing microbial communities within the rhizosphere and endorhizosphere. The findings showed that inoculation with the R3 strain considerably increased the amounts of nitrate nitrogen, ammonium nitrogen, and available phosphorus in the rhizosphere by 14.77%, 27.83%, and 22.67%, respectively, in comparison to the control (CK). Additionally, the theoretical yield of rice was enhanced by 8.81% due to this inoculation, primarily through a 10.24% increase in the effective number of rice panicles and a 4.14% increase in the seed setting rate. Further analysis revealed that the structure of the native nitrogen-fixing microbial communities within the rhizosphere and endorhizosphere were altered by inoculation with the R3 strain, significantly increasing the α-diversity of the communities. The relative abundance of key nitrogen-fixing genera such as *Ralstonia*, *Azotobacter*, *Geobacter*, *Streptomyces*, and *Pseudomonas* were increased, enhancing the quantity and community stability of the nitrogen-fixing community. Consequently, the nitrogen-fixing capacity and sustained activity of the microbial community in the rhizosphere soil were strengthened. Additionally, the expression levels of the nitrogen absorption and transport-related genes *OsNRT1* and *OsPTR9* in rice roots were upregulated by inoculation with the R3 strain, potentially contributing to the increased rice yield. Our study has revealed the potential microbial mechanisms through which inoculation with nitrogen-fixing bacteria enhances rice yield. This finding provides a scientific basis for subsequent agricultural practices and is of critical importance for increasing rice production and enhancing the ecosystem services of rice fields.

**Supplementary Information:**

The online version contains supplementary material available at 10.1007/s44307-024-00038-4.

## Introduction

Rice (*Oryza sativa* L.), as one of the crucial global staple crops, has yields and quality that are vital for food security (Muthayya et al. 2014). Chemical fertilizers, traditionally used to enhance rice yields, significantly boost growth rate and yield when inorganic nutrients such as nitrogen, phosphorus, and potassium are administered (Iqbal et al. 2020). Nitrogen is a particularly important nutrient, serving as one of the essential nutrients during rice growth (Chen et al. 2023). The proper application of nitrogen can increase chlorophyll content and enhance photosynthesis, thereby improving yields (Gai et al. 2017). However, the excessive use of chemical fertilizers can lead to environmental pollution and soil degradation (Guo et al., 2022; Ramzan et al. 2022). To enhance rice yields and reduce reliance on chemical fertilizers, scientists are continuously exploring biotechnological methods to improve crop productivity. Among these methods, nitrogen-fixing bacteria that convert atmospheric nitrogen into a form usable by plants have demonstrated immense potential in improving soil fertility and promoting crop growth (Banik et al., 2019; Bhattacharjee et al. 2008; Pankievicz et al. 2019).

Nitrogen-fixing bacteria convert atmospheric nitrogen (N_2_) into a form that can be utilized by plants (e.g. ammonia, NH_3_), a process known as biological nitrogen fixation. Their role in nature is extremely important, especially in agroecosystems, where they have a positive impact on the environment by increasing soil fertility and reducing the need for chemical fertilizers (Peoples et al. 1995). Widely recognized as efficient and adaptable microbial resources, nitrogen-fixing bacteria are currently the focus of international and domestic attention for their application in sustainable agricultural production (Reis and Teixeira 2015). Research indicates that these bacteria not only enhance crop yields through nitrogen fixation (Shameem et al., 2023), but also establish symbiotic or mutualistic relationships with crop root systems. Their interactions enhance the secretion of growth hormones in the roots, thereby facilitating root expansion and nutrient absorption, which improves the overall nutritional status and stress resilience of the crops (Islam et al. 2013). Further research has demonstrated that nitrogen-fixing bacteria possess the capability to enhance the soil microenvironment. Through the secretion of growth-regulating substances, enhancement of the soil microenvironment, and suppression of pathogenic microbes, these bacteria indirectly promote rice growth and enhance crop health (Hayat et al. 2010; Romaniuk et al. 2011). These advantages render nitrogen-fixing bacteria a crucial component of sustainable agriculture, capable of enhancing crop yields while mitigating negative environmental impacts. Therefore, the incorporation of these organisms into practical rice cultivation represents a viable strategy.

The complex and diverse microbial communities of soil environments have many unidentified functions, making it challenging to ascertain their impact on soil nutrients and plant growth (Edwards et al. 2015; Peiffer et al. 2013). Nitrogen fixation is catalyzed by the nitrogenase complex, which consists of several proteins, the most vital of which is the ferritin subunit encoded by the *nifH* gene (Angel et al. 2018). Widely employed as a molecular marker, the *nifH* gene is used to characterize nitrogen-fixing microbial communities across various habitats (Hsu and Buckley 2008; Zehr et al. 2003). Through this gene, identifying an accessible nitrogen-fixing bacterium in soil and plant ecosystems could facilitate a deeper exploration of the overall situation of nitrogen-fixing microbial communities in these environments. At the present time, research on nitrogen-fixing bacteria has predominantly focused on their functional roles, with comparatively less attention paid to the structure and interactions within these communities. Conducting further research in these areas could enhance understanding of the mechanisms by which nitrogen-fixing bacterial communities promote rice growth.

To reveal the potential microbial mechanisms by which inoculation with nitrogen-fixing bacteria enhance rice yield, this study utilized an endogenous nitrogen-fixing bacterium isolated from rice seeds in a potted soil cultivation experiment. The effects of exogenously added nitrogen-fixing bacteria on nutrients in the rhizosphere soil and on rice growth were investigated. Furthermore, the nitrogen-fixing microbial communities within the rhizosphere and the roots themselves were analyzed following inoculation. Overall, this study aims to: (1) analyze changes in nutrient content within the rice rhizosphere and plant growth following inoculation with bacterial strains; (2) explore the responses of nitrogen-fixing microbial communities within the rice rhizosphere and roots to inoculation of nitrogen-fixing bacteria; (3) elucidate the mechanisms through which the inoculation of nitrogen-fixing bacteria enhance rice yield. This study's findings are significantly important for enhancing rice production and improving the ecosystem services of rice fields. They provide a scientific basis for agricultural practices and offer new insights and methods to promote sustainable agricultural development and ensure food security.

## Materials and methods

### Description of rice varieties, bacterial cultures, and soils

“Huang Huazhan”, a conventional rice variety, was provided by the Hunan Provincial Academy of Agricultural Sciences for testing. Well-filled rice seeds were selected, soaked in a 1–2% potassium permanganate solution for 24 h, and subsequently rinsed thoroughly with distilled water. After soaking in clear water for 2–3 days, the cleaned seeds were spread out in a germination tray and placed in a 30 °C incubator to promote germination. At the three-leaf stage, rice seedlings with similar size and vigor were selected for a soil-based pot experiment. The endophytic nitrogen-fixing bacterium R3 (*Herbaspirillum*), previously isolated from “Huang Huazhan” rice seeds by the research group, was used in the tests. The strain’s specific physiological functions are detailed in Table S1. The R3 strain was inoculated at a 3% rate into 300 mL of 1/2 LB liquid medium and incubated on a shaker (28 °C, 180 rpm) for 36 h. Following this, the culture underwent low-temperature high-speed centrifugation (4 °C, 6000 rpm, 10 min) and the pelleted bacterial cells were subsequently resuspended in 0.85% KCl solution. Using a cell counting plate under a light microscope, the concentration was measured, and the suspension was subsequently adjusted to a concentration of 1 × 10^8^ cfu·mL^−1^ for future use. The Hunan Rice Research Institute supplied the potting soil for the experiments, and its basic chemical properties detailed in Table S2.

### Pot experimental design

Soil-cultivated rice pot experiments were conducted in the greenhouse facilities of Hunan Agricultural University, where daytime temperatures ranged from 29 °C to 40 °C and nighttime temperatures ranged from 24 °C to 29 °C, with relative humidity between 65 and 85%. The soil cultivation containers, made of polyethylene, had a top diameter of 40 cm, a bottom diameter of 35 cm, and a height of 30 cm, each containing 15 kg of test soil. Rice seedlings at the three-leaf stage were transplanted into the containers. Following the greening of the transplanted rice, inoculation with the bacterial strain was performed at the rhizosphere (roughly one week after transplanting). Two treatments were included in the experiment: (1) CK: a blank control group without bacterial inoculation, to which an equivalent amount of distilled water was added; (2) R3: treatment with 50 mL of R3 bacterial suspension at a concentration of 1 × 10^8^ cfu·mL^−1^. Rice cultivation in each treatment involved 5 pots, with 3 plants per pot and 2 rice seedlings per plant. Water and fertilizer management followed standard field practices, and the rice was cultivated to maturity.

### Rice plant and soil sampling

At the rice maturity stage, 10 plants were selected randomly from each treatment. The plants were thoroughly rinsed with distilled water and then divided into roots, stems, leaves, and panicles. The roots, stems, and leaves were placed in clean envelopes, initially dried in an oven at 105℃ for 30 min, and then further dried at 80℃ until they reached a constant weight. The grains were sun-dried. The number of effective panicles per plant, grains per panicle, thousand-grain weight, and seed-setting rate were recorded. Additionally, the weight of the dry matter for each plant part was recorded. The samples were ground into powder for further processing to determine the nitrogen content of the various plant parts. An additional portion of fresh rice roots, stem, leaf and grain from each treatment were thoroughly rinsed with distilled water and then immersed in 5% NaClO for 3 min, and then immersed in 75% ethanol for 5 min to disinfect the surface. Residual chemicals were removed by repeatedly rinsing the roots with sterile distilled water. The cleaned roots were stored at -80℃ for later testing. Simultaneously, rhizosphere soil samples corresponding to each treatment were divided into two portions: one portion was air-dried indoors, sieved through a 100-mesh screen, and used for analysis of chemical properties; the remaining soil was stored at -80℃ for DNA extraction and high-throughput sequencing.

### Measurement of related indices of rice plant and soil chemical properties

An electronic balance was used to weigh the dry matter for each section of the rice plants (BSA2202, Sartorius, Germany). The theoretical yield per plant was calculated by multiplying the number of effective panicles per plant by the number of grains per panicle and the thousand-grain weight, and dividing the result by 1000. Using the potentiometric approach, the pH of the soil was measured with a pH meter (FE28, Mettler Toledo, Switzerland) (Cossel et al. 2019). The total nitrogen (TN) content in rice plants and soil was determined using an elemental analyzer (CN802, Shanghai, China). The available phosphorus (AP) content in soil was measured with a UV–Vis spectrophotometer (LAMBDA1050, PerkinElmer, USA) (Murphy and Riley 1962). Atomic emission spectroscopy (ICP-AES) using inductively coupled plasma was used to determine the amount of available potassium (AK) in the soil samples (Ramsey and Thompson 1987). Using 0.01 mol·L^−1^ CaCl_2_, soil nitrate nitrogen (NO_3_-N) and ammonium nitrogen (NH_4_-N) were extracted, and a continuous flow analyzer (FLOWSTAR2020, Shanghai, China) was used for analysis (Gómez-Brandón et al., 2015).

### Detection of *nifH* gene abundance and microbial communities in rhizosphere and root

A 0.5 g sample of rhizosphere soil was thoroughly ground in liquid nitrogen, followed by DNA extraction using a soil genomic DNA extraction kit (Norgen Biotek, Canada). Similarly, a 0.2 g sample of rice roots was thoroughly ground in liquid nitrogen, and a plant genomic DNA extraction kit (Norgen Biotek, Canada) was used to extract the DNA. The DNA was assessed for purity and concentration using a Thermo NanoDrop One spectrophotometer. PCR amplification was performed using genomic DNA as the template and the primer pair *nifH*-F (AAAGGYGGWATCGGYAARTCCACCAC) and *nifH*-R (TTGTTSGCSGCRTACATSGCCATCAT) (Li et al. 2023a). The PCR products' length and concentration were confirmed by 1% agarose gel electrophoresis. Qualified products were then sequenced on the Illumina NovaSeq 6000 platform using PE250 (Erlich et al. 2008; Cock et al., 2010). Quantitative polymerase chain reaction (qPCR) was used to determine the *nifH* gene copy number (Church et al. 2005). The prepared reaction system, consisting of the extracted nucleic acid template, specific primers for *nifH*, dNTPs, thermostable DNA polymerase, buffer, and fluorescent dye, was placed in a real-time quantitative PCR instrument (7700, Thermo Fisher Scientific, USA) and subjected to cycling under predetermined temperature and time conditions. During each cycle's extension or annealing/extension phase, the real-time quantitative PCR instrument monitored the fluorescence signal intensity continuously. The threshold cycle number (Ct value) was determined from the changes in fluorescence signal intensity as the cycle numbers increased. Using a standard curve created from known amounts of standards, the copy number of the *nifH* gene in the samples was determined.

### Detection of nitrogen uptake and transport-related gene expression in rice

Rice tissue samples from different parts of the plants were removed from a -80℃ freezer. An appropriate amount of each sample was ground into a fine powder with a small amount of liquid nitrogen using a sterile mortar and pestle that had been pre-cooled with liquid nitrogen. Approximately 50 mg of the ground sample was transferred into a 1.5 mL EP tube. RNA was extracted with a plant RNA extraction kit (Sangon Biotech, China). The extracted RNA samples were quickly thawed on ice, treated to remove gDNA, and reverse transcribed. The resulting cDNA was stored at -20℃ for subsequent experiments. Specific primers for the reference gene *eEF-2* and the nitrogen uptake and transport-related genes *OsNRT1* and *OsPTR9* were designed (Table S3). The 2 × SuperReal PreMix Plus, template, forward and reverse primers, and RNase-Free ddH_2_O required for qPCR were thawed on ice. The reaction system was prepared on ice, with all reagents gently mixed at room temperature and briefly centrifuged to ensure all components settled at the bottom of the tubes and no air bubbles remained. The reaction was then initiated by inserting the reaction system into a real-time quantitative PCR device. After the reaction, Ct values were recorded. The gene *eEF-2* was used as the reference, and the average Ct value was used as the reference gene's Ct value. The values of the fluorescence curve and the Ct values were used to compute the target genes' relative expression levels. The fold change between the two was defined as 2^−ΔΔCt^, where ΔCt = Ct (target gene) – Ct (reference gene) (De Wilde et al. 2014; Livak and Schmittgen 2001).

### Data processing and analysis

The differences in various parameters between treatments were analyzed through T-tests using SPSS 19.0, with significance determined at *P* < 0.05. Data reported in this paper are mean ± standard error (SE) of ten replicates.

Using Cutadapt (https://github.com/marcelm/cutadapt/), primers were removed from the raw sequences, and then forward and reverse sequences were merged using FLASH (Magoc and Salzberg 2011). UPARSE was used to cluster merged sequences into Operational Taxonomic Units (OTUs) at a 97% similarity level (Edgar 2013), and these were classified with a minimum confidence level of 50% using the RDP classifier (Wang et al. 2007). Using the Vegan package in R, the alpha diversity, beta diversity, and species abundance composition of nitrogen-fixing microbial communities were examined (3.4.4). Differences in bacterial community composition between treatments were also tested by statistical analyses of similarity (ANOSIM, MRPP and PERMANOVA) between groups. The number of shared and unique OTUs across all samples was determined using Venn analysis (Mo et al. 2021).

The random matrix theory (RMT)-based Molecular Ecological Network Analysis Pipeline (MENAP) at http://ieg4.rccc.ou.edu/mena was used to examine genetic correlation networks (Deng et al. 2012). For further computations, only OTUs that were present in 70% of the samples were retained. Network visualization and topological feature calculations were conducted with Gephi software (Jacomy et al. 2014; Shi et al. 2016). The principal discriminative bacterial taxa among treatments were determined using the Linear Discriminant Analysis (LDA) Effect Size (LEfSe) approach (LDA score > 3.0, *P* < 0.05).

The associations between bacterial species in the rhizosphere and roots and environmental variables were assessed using Pearson correlation. Mantel analysis using a publicly available pipeline (http://mem.rcees.ac.cn:8080/), was performed to ascertain the correlations between microbial communities in the rhizosphere and roots, environmental factors, nitrogen uptake and transport-related gene expression in rice roots, theoretical yield, and yield components (Yu et al. 2021b).

Using the RandomForest package in R, a random forest (RF) analysis was performed to determine the major taxonomic variables impacting the nitrogen and ammonium content of soil nitrate (Liaw and Wiener 2002). Using the PLSPM package in R (Michel et al. 2005), a partial least squares path modeling (PLS-PM) analysis was carried out to investigate the cascade of interactions among rice yield, the alpha diversity of bacterial communities in the rhizosphere and roots, key bacterial taxa in the rhizosphere and roots, nitrogen uptake and transport-related gene expression in rice roots, and ammonium and nitrate nitrogen content in the rhizosphere soil. The model was comprised of theoretically-driven latent variables that were represented by a set of observable indicators. Path coefficients represent direct impacts, whereas the product of path coefficients present indirect effects. The sum of the direct and indirect effects was used to compute the overall effect (Yu et al. 2021a).

## Results

### Chemical characteristics of rhizosphere soil and plant growth dynamics in rice

As indicated in Table 1, following inoculation with the nitrogen-fixing bacterium R3, the concentrations of NO_3_-N, NH_4_-N, and AP in the rice rhizosphere soil were found to increase significantly, by 14.77%, 27.83%, and 22.67%, respectively, compared to the CK (*P* < 0.05). However, no significant changes were observed in pH, TN, and AK content. These findings imply that inoculation with the R3 strain facilitates the transformation of readily available nitrogen and phosphorus in the soil.

**Table 1:** Rice rhizosphere soil chemical properties and theoretical yield of rice and its constituent factors

Treatment		CK	R3
Rhizosphere Soil chemical properties	pH	7.07±0.03a	7.10±0.04a
	TN/g·kg^-1^	1.60±0.12a	1.61±0.12a
	NO_3_-N/mg·kg^-1^	14.49±0.69b	16.63±1.11a
	NH_4_-N/mg·kg^-1^	4.60±0.37b	5.88±0.43a
	AP/mg·kg^-1^	10.32±1.03b	12.66±1.25a
	AK/mg·kg^-1^	38.21±0.55a	39.17±0.58a
Theoretical yield of rice and its constituent factors	Effective panicle number/ panicle·plant^-1^	16.60±1.17b	18.30±1.06a
	Average grain number per panicle/grain	116.00±8.47a	114.10±4.73a
	Thousand grain weight/g	21.53±0.10a	21.55±0.17a
	Seed setting rate/％	78.90±1.08b	82.17±1.27a
	Theoretical yield/g·plant^-1^	41.31±2.13b	44.95±2.39a

Mean values (± S.D., *n* = 10) among CK and R3 treatments based on Duncan test. Different letters indicate significant differences (*P* < 0.05)

The rice plants' dry weight and nitrogen buildup in different sections were considerably higher after being inoculated with nitrogen-fixing bacterium R3 as opposed to the CK (Table S4, Fig. S1) (*P* < 0.05). As shown in Table 1, inoculation with the R3 strain significantly increased the theoretical yield per rice plant by 8.81% compared to the control (*P* < 0.05). Subsequent analysis of the factors contributing to the theoretical yield per plant indicated that significant increases in the number of effective panicles per plant and the seed-setting rate were the principal drivers of enhanced rice yield (*P* < 0.05). Specifically, increases of 10.24% in the number of effective panicles and 4.14% in the seed-setting rate were observed. These findings suggest that the primary enhancement of rice yield through inoculation with the R3 strain is attributable to increases in the number of effective panicles and the seed-setting rate.

### Diversity, nifH gene abundance, structure, and composition of nitrogen-fixing *bacteria*

**Fig. 1:**
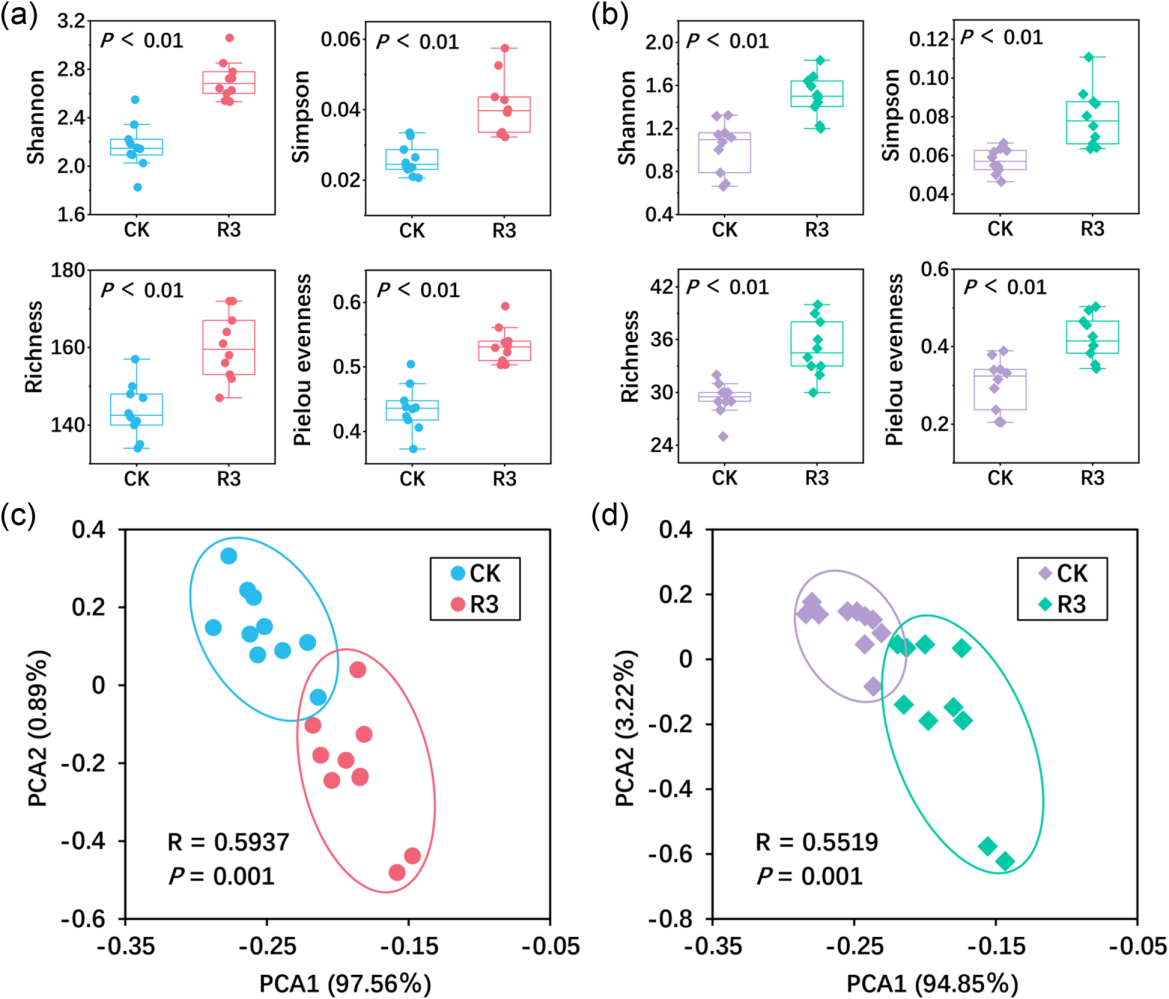
The α-diversity of the *nifH* gene microbial community was assessed in the rice rhizosphere soil (**a**) and roots (**b**) under each treatment; A principal coordinate analysis was conducted on the *nifH* gene microbial community in the rice rhizosphere soil (**c**) and roots (**d**)

From each soil and rice root sample, an average of 36,000 sequences were obtained. The rarefaction curves of the observed OTUs demonstrated good sequencing depth and coverage (Fig. S2). All sequences detected in soil samples were assigned to 260 OTUs after trimming, whereas those from rice root samples were assigned to 85 OTUs. The Shannon, Simpson, Pielou evenness, and Richness indices revealed that the α-diversity of nitrogen-fixing microbial communities both in the rhizosphere soil and within the roots of rice treated with R3 were significantly higher than those observed in the CK treatment (*P* < 0.05) (Fig. 1a, b). Furthermore, the comparative abundance of the *nifH* gene in the R3 and CK treatments was examined. In the R3 treatment, *nifH* gene abundance in the rice rhizosphere soil was substantially higher than the CK treatment's (*P* < 0.05), but there was no discernible difference in the *nifH* gene abundance between the roots of the two treatments (Fig. S3). PCA analysis results showed that samples from both the rhizosphere soil and the roots formed discrete clusters that aligned with the R3 and CK treatments (Fig. 1c, d). Furthermore, analyses using the Bray–Curtis matrix (Table S5) through three non-parametric tests (MRPP, PERMANOVA, and ANOSIM) consistently revealed significant structural differences in the nitrogen-fixing microbial communities between the R3 and CK treatments, both in the rhizosphere soil and within the roots (*P* < 0.05). This result implies that the structures of the nitrogen-fixing microbial communities in the rice roots and the rhizosphere soil were considerably changed by inoculation of the R3 strain.

**Fig. 2:**
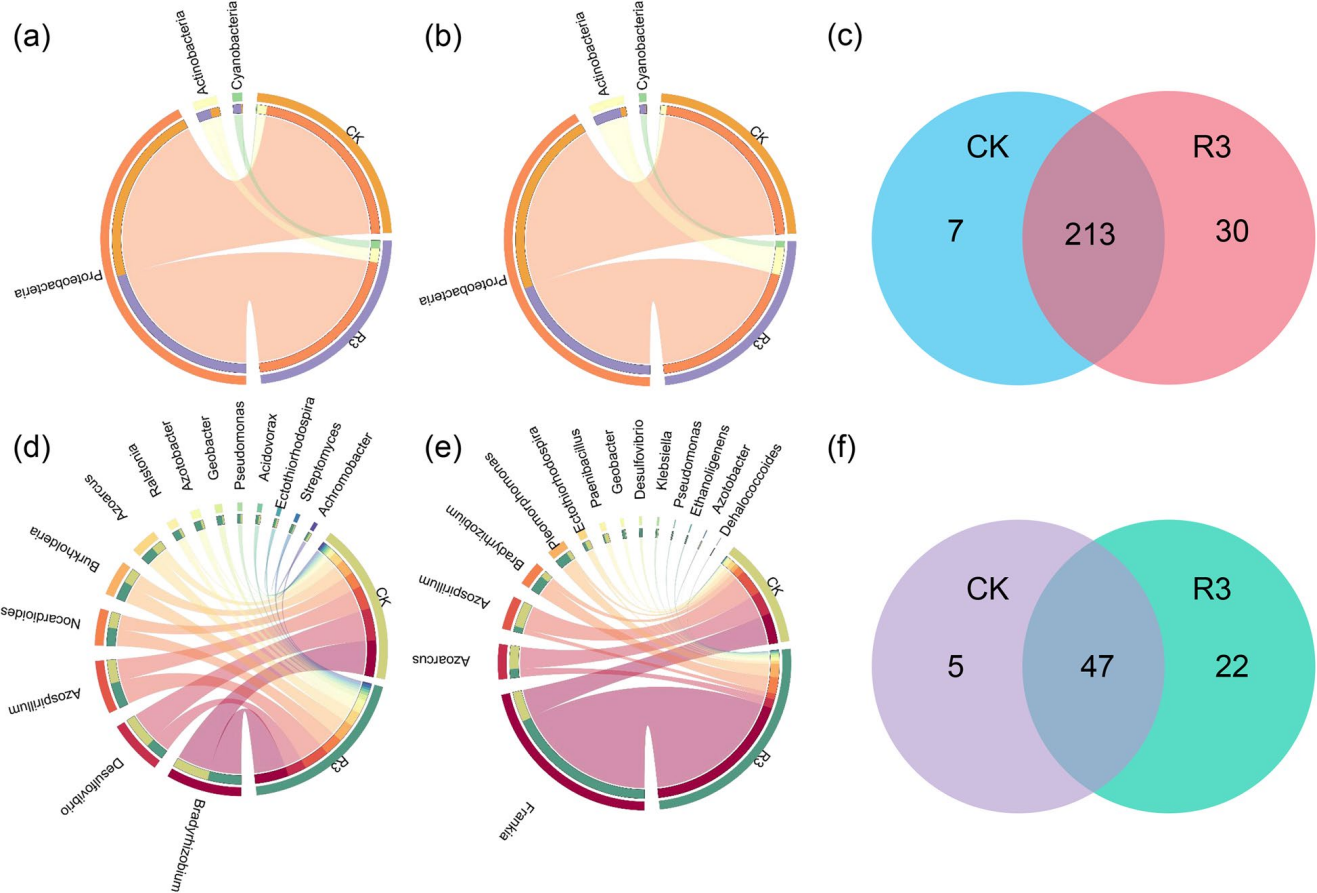
The composition of the *nifH* gene microbial community was analyzed for the rice rhizosphere soil (**a**) and roots (**b**) at the phylum level. Similarly, analyses were conducted at the genus level in rice rhizosphere soil (**d**) and roots (**e**). The Venn diagram displays the number of common and unique OTUs present in the rhizosphere soil (**c**) and roots (**f**) for each treatment

The relative abundances of nitrogen-fixing bacteria in rice rhizosphere soil and roots were altered (Fig. 2a, b, d, e). At the phylum level, the relative abundance of Proteobacteria was significantly reduced by the R3 treatment, while the relative abundances of Actinobacteria and Cyanobacteria were significantly increased in both the rhizosphere soil and the roots compared to the CK treatment (*P* < 0.05) (Fig. S4, S5). At the genus level in the rhizosphere soil, the relative abundance of *Desulfovibrio* was significantly reduced by the R3 treatment compared to the CK treatment, while the relative abundances of *Ralstonia*, *Azotobacter*, *Geobacter*, *Streptomyces*, and *Pseudomonas* were significantly increased (*P* < 0.05) (Fig. S4, S5); Within the roots, the relative abundances of *Azoarcus* and *Azospirillum* were significantly reduced by the R3 treatment compared to the CK treatment, while the relative abundance of *Frankia* was significantly increased (*P* < 0.05) (Fig. S4, S5).

Furthermore, Venn diagram analysis revealed that the nitrogen-fixing microbial communities in rice rhizosphere soil possessed a greater number of shared OTUs. In the rhizosphere soil, 30 unique OTUs were identified in the R3 treatment, whereas only 7 were observed in CK (Fig. 2c). Within the roots, 22 unique OTUs were found in the R3 treatment, whereas only 5 were observed in the CK treatment (Fig. 2f). This indicates that inoculation with the R3 strain not only altered the relative abundances of nitrogen-fixing bacterial groups at both the phylum and genus levels in the rhizosphere soil and within the root but also increased the number of unique OTUs in these environments.

### Network topology and specific bacterial combinations differences of nitrogen-fixing microbial communities

**Fig. 3:**
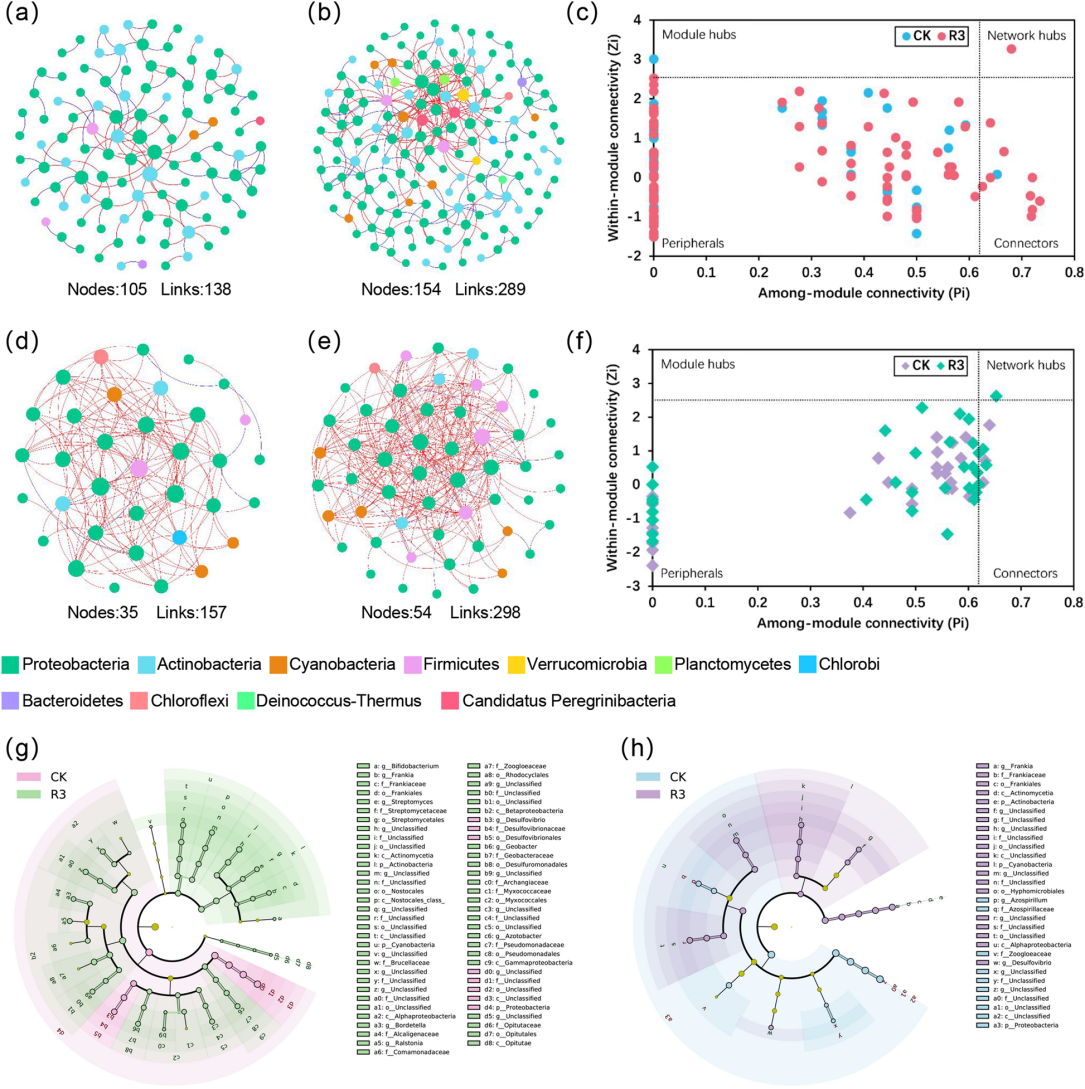
The networks of the *nifH* gene microbial community for rhizosphere soil, CK (**a**) and R3 (**b**) treatments, and rice roots, CK (**d**) and R3 (**e**), were analyzed at the phylum level. Different colors are used to represent the various phyla. The size of each node, representing an OTU, is proportional to its degree. A blue line denotes a positive correlation, whereas a red line signifies a negative correlation. The Zi-Pi diagram shows the distribution of OTUs, based on their network topology in rhizosphere soil (**c**) and roots (**f**), to identify key taxa. Thresholds for Zi and Pi, used to classify OTUs, were set at 2.5 and 0.62, respectively. In the rhizosphere soil (**g**) and root (**h**), Linear discriminant analysis (LDA) effect size (LEfSe) (LDA > 3.3, *P* < 0.05) revealed significant (pink, green, blue, purple) and non-significant (yellow) discriminant classification nodes. The diameter of each circle is proportional to the associated number of taxa. Each concentric ring represents a classification level, progressing from the center to the periphery: domain, phylum, class, order, family, and genus

To determine if R3 strain inoculation influenced the interactions among nitrogen-fixing bacteria within the rhizosphere soil and roots of rice, the corresponding interaction networks were constructed to elucidate their responses (Fig. 3a, b, d, e). In both the rhizosphere soil and roots, networks from the R3 treatment, relative to CK, demonstrated increased total nodes and links, higher average degree (avgK), and a shorter average path distance (Table S6). Several network topology indicators consistently demonstrated that the networks of nitrogen-fixing microbes in the rhizosphere soil and roots of rice inoculated with the R3 strain exhibited greater complexity compared to those in CK.

The potential topological roles of each OTU within the nitrogen-fixing bacterial networks across the four sample groups were identified through RMT-based network analysis (Fig. 3c, f). Based on their among-module (Pi) and within-module (Zi) connection values, nodes were classified into four types: peripherals, connectors, module hubs, and network hubs. Connectors, module hubs, and network hubs were considered key taxonomic units (Deng et al. 2012). In the rice rhizosphere soil, key taxonomic units in the CK treatment included OTU_113 and OTU_193, while the R3 treatment featured OTU_10, OTU_88, OTU_114, OTU_125, OTU_140, OTU_149, OTU_162, OTU_166, OTU_185, OTU_211, and OTU_215. Within the roots, the key taxonomic units for the CK treatment were OTU_55, OTU_57, OTU_63, and OTU_75, while those in the R3 treatment included OTU_29, OTU_38, OTU_66, and OTU_82 (Table S7). This suggests that inoculation with the R3 strain led to an increase in the number of key nitrogen-fixing bacterial taxa within the rhizosphere soil network.

LEfSe analysis was used to identify specific bacterial compositions within each treatment. In the rhizosphere soil of rice, a total of 65 distinct taxonomic groups were identified (Fig. 3g), 57 of which were specific to the R3 treatment's nitrogen-fixing bacterial groups. These groups were predominantly comprised of Cyanobacteria, Actinobacteria, Verrucomicrobia, Firmicutes, and Proteobacteria. Conversely, only 8 nitrogen-fixing bacterial groups specific to the CK treatment were identified, all belonging to Proteobacteria. Within the rice roots, 30 distinct taxonomic groups were identified (Fig. 3h), with 20 specific nitrogen-fixing bacterial groups attributable to the R3 treatment. These groups primarily consisted of Cyanobacteria, Actinobacteria, and Proteobacteria. In contrast, only 10 specific nitrogen-fixing bacterial groups, all belonging to Proteobacteria, were identified as part of the CK treatment. Overall, the specific nitrogen-fixing bacterial groups in the rhizosphere soil and roots of rice inoculated with the R3 strain were found to be more numerous and diverse than those associated with CK. These compositional differences may contribute to variations in soil chemical properties and rice growth.

### Relative expression of nitrogen uptake and transport genes in rice plant tissues

**Fig. 4:**
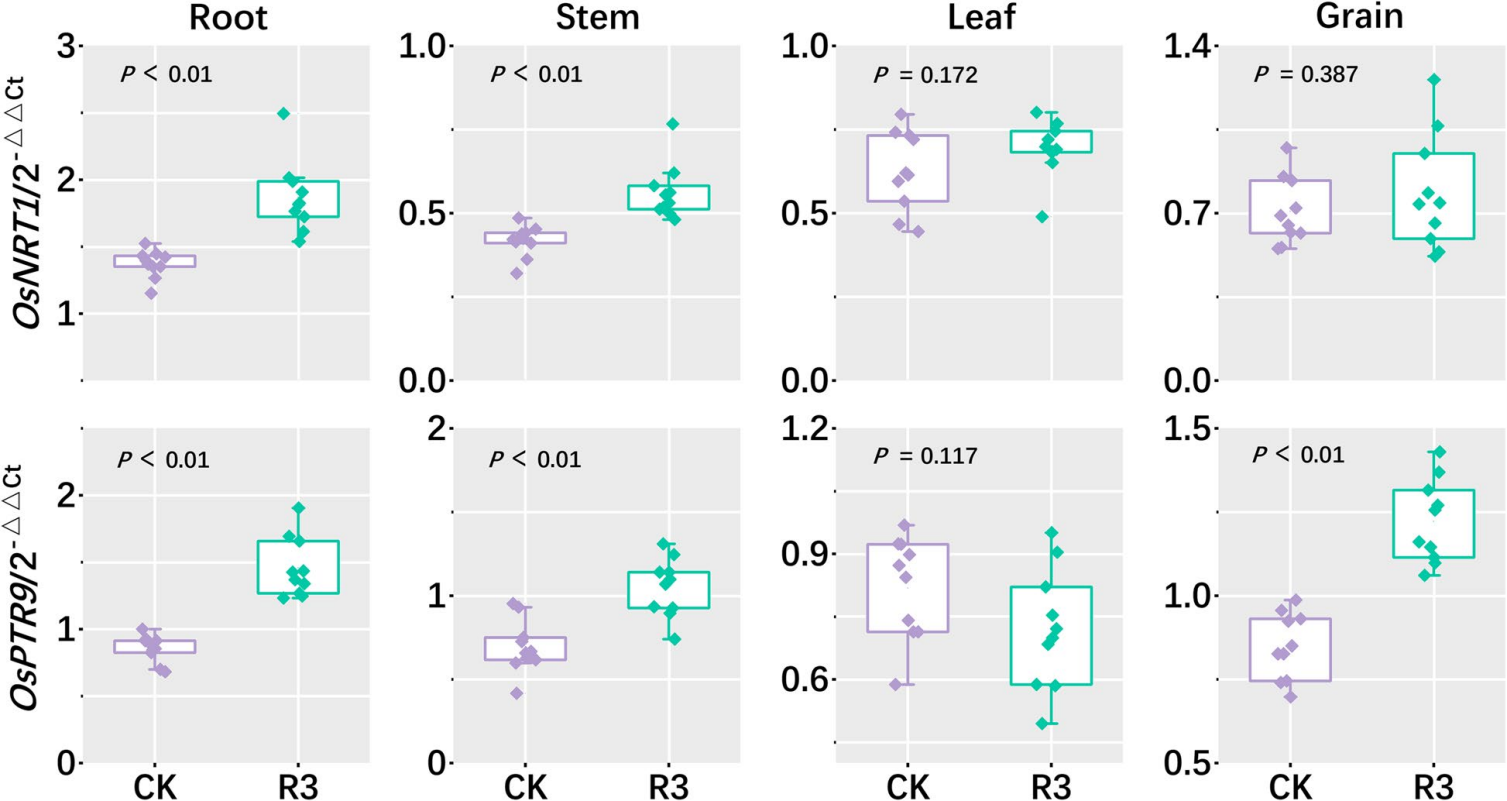
The relative expression levels of *OsNRT1* and *OsPTR9* genes were analyzed in different parts of rice plants. The *P* value signifies the level of significance between two groups of data; *P* < 0.05 is considered significant, while *P* < 0.01 is considered extremely significant

Using fluorescence quantitative PCR, the expression of genes associated with nitrogen uptake transporters (*OsNRT1*, *OsPTR9*) in different tissues of the rice plant were measured to evaluate the reaction of the rice plant itself to inoculation with the R3 strain (Fig. 4). Results showed that expression levels of *OsNRT1* and *OsPTR9* in the roots exceeded those in the stem sheaths, leaves, and brown rice. Inoculation with the R3 strain was found to significantly upregulate the expression levels of the genes *OsNRT1* and *OsPTR9* in the roots and stems of rice plants. At the same time, the expression of gene *OsPTR9* was also significantly elevated in brown rice. No significant changes in gene expression were observed within the leaves. Changes in the expression of these genes may affect the efficiency of nitrogen utilization by rice in the rhizosphere environment, with alterations in root-related gene expression possibly being of greatest significance.

### Correlations among nitrogen-fixing microbes, environmental factors, and rice indices

**Fig. 5:**
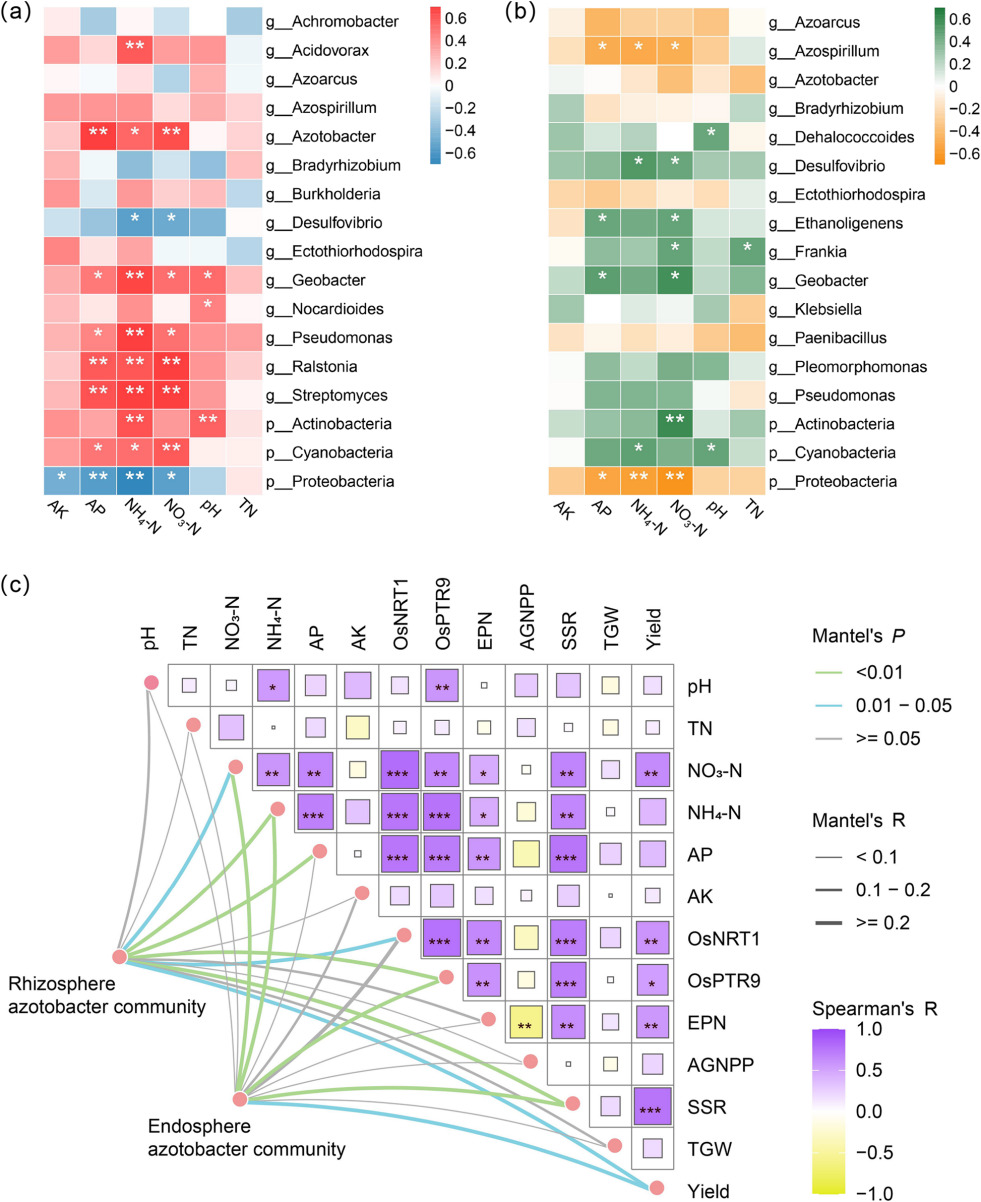
A correlation heat map was created to depict the associations between the soil environmental factors and nitrogen-fixing bacterial groups in rice rhizosphere soil (**a**) and roots (**b**). A Pearson’s correlation matrix, illustrated by the square diagram, depicts the associations between soil factors. Mantel test (**c**) was used to detect relationships between nitrogen-fixing bacterial communities in rice rhizosphere soil, roots, and various environmental factors and rice-related indicators. EPN: Effective panicle number, AGNPP: Average grain number per panicle, TGW: Thousand grain weight, SSR: Seed setting rate. The Pearson correlation coefficient (r) is represented by hue. Asterisks in the grid and square plots indicate significant Pearson correlation: *, *P* < 0.05; **, *P* < 0.01; ***, *P* < 0.001

A substantial link was found by Pearson correlation analysis between the relative abundance of nitrogen-fixing bacterial communities in the rhizosphere soil and the roots of rice, and soil environmental variables (Fig. 5a, b). The results showed that the most diverse groups of nitrogen-fixing bacterial taxa associated with AK, NH_4_-N and NO_3_-N contents were found in the rhizosphere soils of rice. There was a substantial negative correlation between the relative abundance of Proteobacteria and AK content, and both AK and NO_3_-N contents substantial positively correlated with the relative abundances of Cyanobacteria, *Streptomyces*, *Ralstonia*, *Pseudomonas*, *Geobacter*, and *Azospirillum*. Furthermore, NH_4_-N and NO_3_-N contents both showed substantial negative correlations with the relative abundances of Proteobacteria and *Desulfovibrio*. NH_4_-N content was significantly positively correlated with the relative abundances of Cyanobacteria, Actinobacteria, *Streptomyces*, *Ralstonia*, *Pseudomonas*, *Geobacter*, *Azospirillum*, and *Acidovorax*. Within rice roots, the most diverse group of nitrogen-fixing bacterial taxa were associated with NO_3_-N content. NO_3_-N content was be substantial negatively correlated with the relative abundance of Proteobacteria and *Azospirillum*, and substantial positively correlations with Actinobacteria, *Geobacter*, *Frankia*, *Ethanoligenens*, and *Desulfovibrio*. These results suggest that there exists a potential correlation between changes in the abundance of soil nitrogen-fixing bacterial taxa in the rhizosphere of rice related to soil AK, NH_4_-N, and NO_3_-N content, and that changes in soil nitrogen-fixing bacterial taxa and soil nutrient content may be one of the factors contributing to the changes in the abundance of nitrogen-fixing bacterial taxa in the root.

Potential associations between soil environmental factors, rice-related indicators, and nitrogen-fixing bacterial communities were revealed through Pearson's correlation matrix and Mantel tests (Fig. 5c). It was found that the levels of NH_4_-N and NO_3_-N, along with the expression of the gene *OsPTR9*, seed setting rate, and rice yield, demonstrate significant correlations with the nitrogen-fixing bacterial communities in the rhizosphere and within the roots of rice (*P* < 0.05). Additionally, it was found that there were substantial positive relationships (*P* < 0.05) between the number of effective panicles, NO_3_-N content, expression levels of the genes *OsNRT1* and *OsPTR9*, seed setting rate, and rice yield (*P* < 0.05). The number of effective panicles and seed setting rate with NH_4_-N content, NO_3_-N content, and the expression levels of *OsNRT1* and *OsPTR9* were also found to have significant positive associations (*P* < 0.05). Additionally, substantial positive correlations were exhibited between the expression levels of *OsNRT1*, *OsPTR9* and AK, NH_4_-N, NO_3_-N content (*P* < 0.05).

### The impact of biological and soil factors on nitrogen fixation and rice yield

**Fig. 6:**
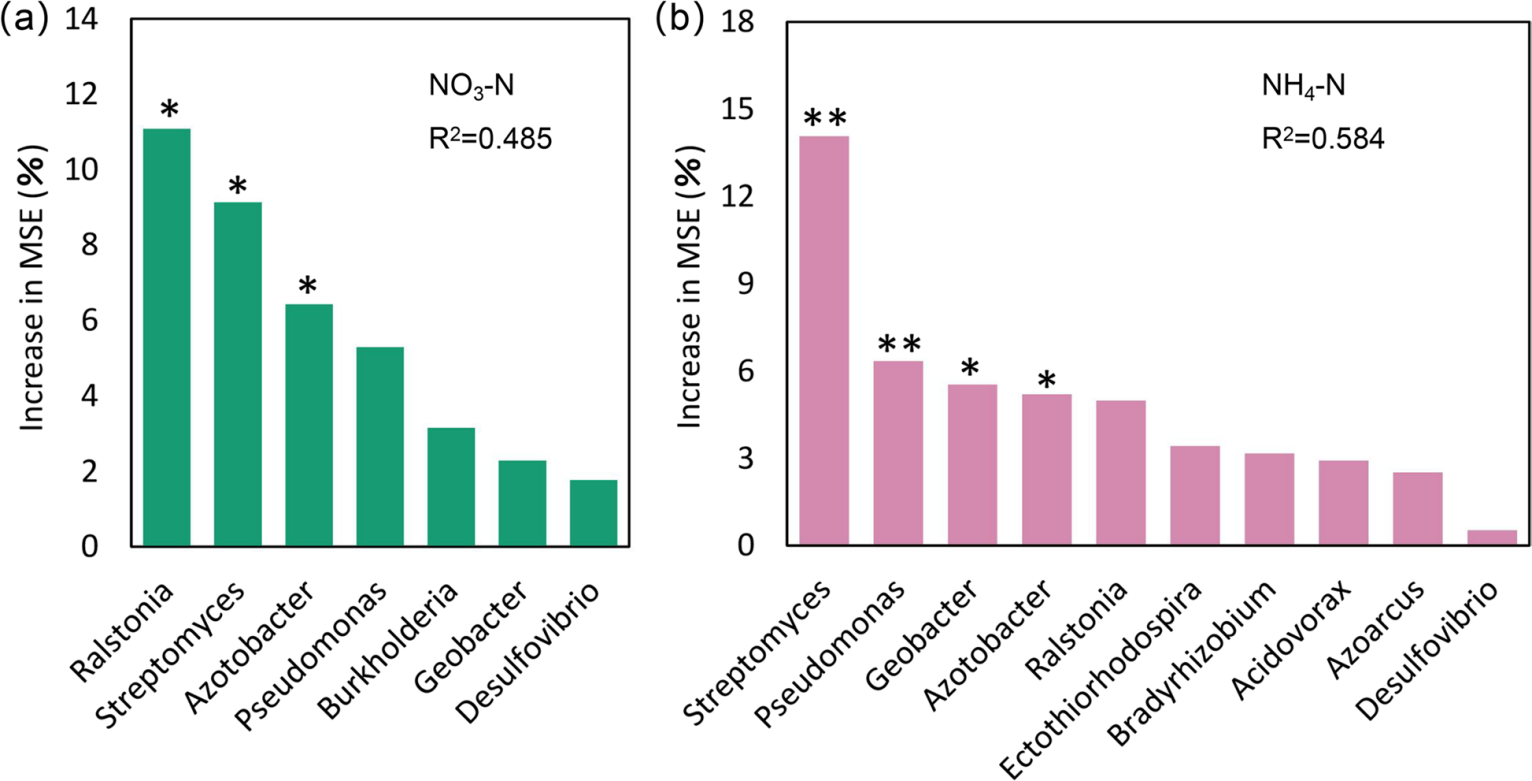
Key taxa predicted to influence nitrate nitrogen content (**a**) and ammonium nitrogen content (**b**) in rice inter-root soils were identified through random forest analysis. The mean square error (MSE) percentage increase of the variables was used to determine the significance of these predictors; larger MSE% values indicate greater importance. *: *P* < 0.05; **: *P* < 0.01

Random forest (RF) models were constructed to assess the biological contribution of inter-root soil nitrogen-fixing bacterial taxa to soil NO_3_-N and NH_4_-N content (Fig. 6a, b). According to the results, *Ralstonia*, *Streptomyces*, and *Azotobacter* were identified as the primary contributors to soil NO_3_-N content. Whereas *Streptomyces*, *Pseudomonas*, *Geobacter*, and *Azotobacter* were identified as the major contributors to soil NH_4_-N content, with significant contributions from *Streptomyces* and *Azotobacter* to both NO_3_-N and NH_4_-N content. These findings suggest that certain key groups may play a crucial role in nitrogen fixation within the rhizosphere soil system.

**Fig. 7:**
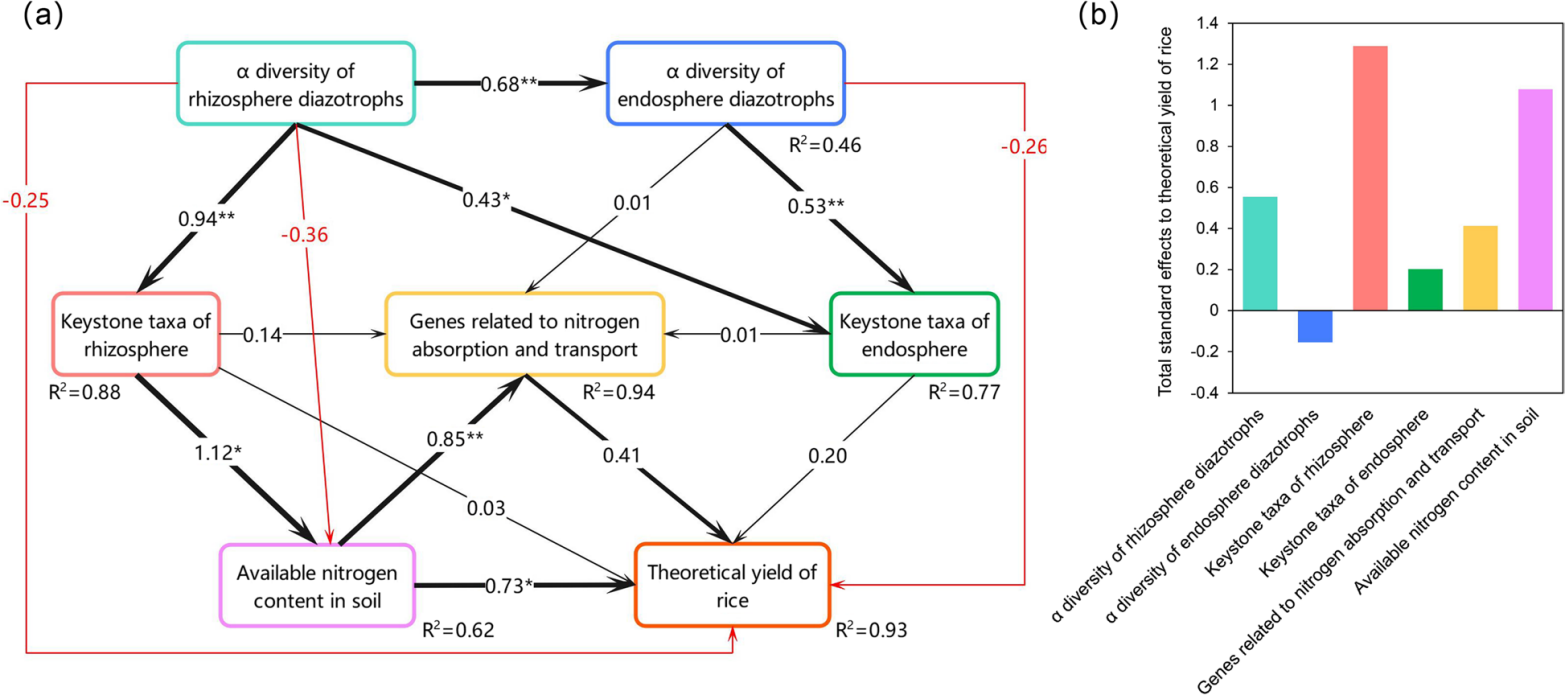
Cascading relationships among biological and soil factors with respect to rice yield were examined. The main pathways of influence of latent variables on rice yield were revealed by the partial least squares path model (**a**) and the standardized effects of factors (**b**). In the model, each long box corresponds to a latent variable, whereas the parameter within each long box denotes an explicit variable. Larger pathway coefficients are indicated by wider arrow lines, while black and red lines denote positive and negative effects, respectively. *: *P* < 0.05; **: *P* < 0.01

A PLS-PM model was constructed to better understand the relative contributions of biological and soil factors to soil nitrogen fixation and rice yield (Fig. 7a, Table S8). Overall, the α-diversity of rhizospheric nitrogen-fixing microbes significantly positively influenced the α-diversity of endophytic nitrogen-fixing microbes, key rhizospheric groups, and key endophytic groups (path coefficients = 0.68, *P* < 0.01; path coefficients = 0.94, *P* < 0.01; path coefficients = 0.43, *P* < 0.05). The α-diversity of endophytic nitrogen-fixing microbes also significantly positively impacted key endophytic groups (path coefficients = 0.53, *P* < 0.01). Key rhizospheric groups had a significant positive effect on soil available nitrogen content (path coefficients = 1.12, *P* < 0.05), which in turn significantly influenced the expression of genes related to nitrogen uptake and transport in rice roots, as well as yield (path coefficients = 0.85, *P* < 0.01; path coefficients = 0.73, *P* < 0.05). Furthermore, the analysis indicated that key nitrogen-fixing groups in the rhizosphere exerted the most significant positive effect on rice yield (standardized effects = 1.29), followed by soil available nitrogen content and the α-diversity of rhizospheric nitrogen-fixing microbes (standardized effects = 1.08; standardized effects = 0.55) (Fig. 7b). These results suggest that the α-diversity of key nitrogen-fixing groups in the rhizosphere, and soil available nitrogen content were the primary drivers in enhancing rice yield.

## Discussion

Nitrogen-fixing bacteria are essential in maintaining the nitrogen cycle within ecosystems, promoting sustainable agricultural development, and protecting the environment (Soumare et al. 2020). The factors for high rice yield are complex and closely related to climate and soil nutrient composition (Katsura et al. 2008; Li et al. 2009). Furthermore, beneficial activities of soil microorganisms, such as improving soil structure, activating nutrients, suppressing pathogen growth through antimicrobial substances and competitive exclusion, and inducing plant systemic resistance, significantly contribute to increased rice production (Hayat et al. 2010; Romaniuk et al. 2011). Extensive research has confirmed the yield-enhancing effects of nitrogen-fixing bacteria in agriculture, establishing the exogenous addition of these bacteria in paddy fields as a valuable management practice in organic farming (Aloo et al. 2022; Zhang et al. 2023).

This study investigated the effects of exogenously adding the nitrogen-fixing bacterium R3 (*Herbaspirillum*) to the rhizosphere soil of rice on soil nutrients and rice yield. Following inoculation with the R3 strain, there was a significant increase in the concentrations of NO_3_-N, NH_4_-N, and AP in the rhizosphere soil, as well as a significant rise in the theoretical rice yield (Table 1), which was positively correlated with NO_3_-N levels (Fig. 5c). The enhancement in rice yield was primarily attributed to an increase in the number of effective panicles and the seed setting rate per panicle. Nitrogen enhances protein synthesis in rice plants, which increases cell division and growth rates, thus facilitating tillering (Huang et al. 2021). Research has demonstrated that nitrogen fertilization significantly increases the number of effective tillers in rice, which in turn boosts the number of panicles and ultimately enhances yield (Wu et al., 2020). Nitrogen, a vital component of chlorophyll, enhances leaf photosynthetic efficiency and increases the accumulation of photosynthetic products when adequately supplied. During the heading and grain-filling stages, these photosynthetic products are transferred to the grains, thereby enhancing rice's seed setting rate (Sun et al. 2012). Consequently, inoculation with the R3 strain enhances yield by increasing available nitrogen in the rhizosphere soil, which promotes rice tillering and improves the seed setting rate per panicle.

However, the mechanisms through which microbial communities enhance rice yield remain to be elucidated. Subsequent analysis of the *nifH* gene-containing microbial communities in the rice rhizosphere soil and roots showed that R3 strain inoculation altered the α-diversity, structure, and composition of these communities (Figs. 1 and 2), and significantly increased the abundance of the *nifH* gene in rhizosphere soil, although no significant change was observed in the roots (*P* < 0.05) (Fig. S3). The *nifH* gene encodes a vital subunit of the nitrogen-fixing nitrogenase enzyme, and high abundance of the *nifH* gene usually indicates the presence of large numbers of active nitrogen-fixing microorganisms, which are capable of fixing atmospheric nitrogen into ammonia and increasing the effective nitrogen content of soil (Reed et al. 2011). Research has demonstrated that the activity of nitrogen-fixing microorganisms can significantly enhance the supply of nitrogen in the soil, thereby promoting plant growth (Mårtensson et al. 2009; Shu et al. 2012). Communities with a high diversity of the *nifH* gene generally implies the presence of a wide range of different nitrogen-fixing microorganisms in the soil that are capable of performing this important process under different environmental conditions, thus increasing the effective nitrogen content of the soil (Feng et al. 2018). The higher diversity of *nifH* genes also helps to maintain the stability of the ecosystem, which is more resilient in the face of environmental changes, thus enabling continuous nitrogen fixation and ensuring a stable supply of effective nitrogen in the soil (Gaby and Buckley 2011; Zehr et al. 2003).

In this study, inoculation with the R3 strain resulted in significant changes in the relative abundance of certain nitrogen-fixing microbial groups in both the rhizosphere soil and the roots of rice. In the rhizosphere soil, compared to CK, a significant increase was observed in the relative abundance of Actinobacteria, Cyanobacteria, *Ralstonia*, *Azotobacter*, *Geobacter*, *Streptomyces*, and *Pseudomonas* following R3 treatment (*P* < 0.05) (Fig. S4). This increase was significantly correlated with the soil NO_3_-N or NH_4_-N content (Fig. 5a). Within the rice roots, compared to CK, the R3 treatment significantly increased the relative abundance of Actinobacteria, Cyanobacteria, and *Frankia* (*P* < 0.05) (Fig. S5), and these changes were also significantly correlated with the soil NO_3_-N or NH_4_-N content (Fig. 5b).

Recent studies have demonstrated that *Ralstonia* exhibits nitrogenase activity under nitrogen-limiting conditions, enabling these organisms to fix nitrogen across various environmental conditions, thus enhancing plant nitrogen supply (Flowers 2023). *Azotobacter, Geobacter*, and *Streptomyces* exhibit strong environmental adaptability, surviving under diverse adverse soil conditions and in anaerobic environments. The nitrogen-fixing capabilities of these microorganisms play a critical role in the soil nitrogen cycle (Li et al. 2023b; Sumbul et al. 2020), Among them, *Streptomyces* also inhibits pathogenic microorganisms in the soil through the secretion of antibiotics and other secondary metabolites, promoting healthy plant growth (Timofeeva et al. 2023). *Pseudomonas* not only enhances the availability of nitrogen in the soil through its nitrogen-fixing actions but also secretes substances that promote plant growth, such as indole-3-acetic acid (IAA) and phosphatases, thereby further enhancing plant nutrient uptake and growth rates (Singh et al. 2023). *Frankia*, which possesses robust nitrogen-fixing capabilities, can form symbiotic relationships with non-leguminous plants (Chaia et al. 2010). Consequently, it can be hypothesized that inoculation with the R3 strain restructures the community of nitrogen-fixing microorganisms within the rice rhizosphere soil and roots, enhancing not only the overall nitrogen-fixing capacity and stability of the soil microbial community but also the nitrogen-fixing ability within the rice root endophytic community, thus increasing the content of available nitrogen in the rhizosphere soil and facilitating absorption and utilization by the rice.

Interactions within nitrogen-fixing microbial communities significantly impact nitrogen fixation. Research indicates that within the plant rhizosphere, elevated diversity and complex network structures of microbial communities enhance nitrogen fixation and utilization efficiency (Kuypers et al. 2018). Furthermore, the selection and introduction of efficient nitrogen-fixing strains enhances the functionality and structural integrity of the nitrogen-fixing microbial network, boosts nitrogen utilization efficiency, and fosters crop growth (Kindler et al., 2022)​. In our study, multiple network topological features (total nodes, total links, average degree (avgK)) consistently indicated (Table S6) that, compared to the CK treatment, the rhizosphere soil and root endophytic compartments of rice inoculated with the R3 strain possessed more core nodes and tightly connected modules. This implies that the connections among nitrogen-fixing microbes in the rhizosphere soil and roots of rice became more complex and interconnected following inoculation with the R3 strain. Corresponding studies have demonstrated that topological features, including an increased number of nodes and links, positively influence network stability, thereby enhancing resistance to environmental changes and ensuring a continuous nitrogen supply (Li et al. 2023c).

Furthermore, to assess the impact of the exogenous nitrogen-fixing bacterium R3 on rice plants, quantitative PCR was employed to measure the expression levels of genes associated with nitrogen uptake and transport (*OsNRT1*, *OsPTR9*) in different parts of the plants. *OsNRT1*, a nitrate transporter in rice, plays a crucial role in the absorption and transport of nitrate (Wang et al., 2018), *OsPTR9* primarily manages the uptake and transport of peptides and amino acids, significantly contributing to the nitrogen metabolism and nutritional balance of plants. Under conditions of high nitrogen, upregulation of *OsPTR9* enhances nitrogen absorption and utilization (Liao et al. 2023). Previous studies have demonstrated that elevated expression of *OsPTR9* enhances nitrogen use efficiency and facilitates effective nitrogen utilization, critically contributing to improvements in both yield and quality of rice (Vera et al., 2021). Our research revealed that expression levels of the genes *OsNRT1* and *OsPTR9* were elevated in the roots of rice compared to the stem, leaves, and brown rice, and that inoculation with the R3 strain significantly upregulated *OsNRT1* and *OsPTR9* in the roots and stem sheaths of the rice plants (Fig. 4), This increased expression was significantly and positively correlated with the number of effective panicles and grains per panicle (Fig. 5c), implying that the substantial upregulation of *OsNRT1* and *OsPTR9* in rice roots contributes to enhanced rice yield.

Exogenous addition of the nitrogen-fixing bacterium R3 influences nitrogen fixation in the rhizosphere soil by modulating the diversity and abundance of key microbial groups, thereby affecting rice yield (Lai et al. 2022; Sepp et al. 2023). As probiotics, nitrogen-fixing bacteria regulate the structure of the microbial community in the rhizosphere, thereby accelerating nutrient transformation in this environment (Gu et al. 2023; Qiu et al. 2023). Our observations indicated that the addition of the exogenous nitrogen-fixing bacterium R3 increased the content of available nitrogen in the rhizosphere soil, potentially contributing to an increase in rice yield. However, the impact of the R3 strain on rice yield likely does not result from a simple single-factor pathway. It is a reasonable assumption, that introducing the exogenous nitrogen-fixing bacterium R3 into the rhizosphere soil modifies the interactions within the native nitrogen-fixing communities, thereby influencing their diversity and structural composition. This alteration results in an increased number and variety of nitrogen-fixing bacteria within the community, enhancing their nitrogen-fixing function and stabilizing their structure. Consequently, this leads to an increase in the content of available nitrogen in the rhizosphere soil, promoting rice tillering and improving the seed-setting rate, thereby enhancing rice yield. Furthermore, the upregulation of genes associated with nitrogen uptake and transport in rice plant roots contributes to an increase in rice yield. In summary, the α-diversity of nitrogen-fixing microbial communities, the presence of key nitrogen-fixing groups, and the levels of available nitrogen in rhizosphere soil play crucial roles in enhancing rice yield.

## Conclusions

Our study demonstrated that exogenous inoculation with the R3 strain significantly increases available nitrogen content in the soil, promoting rice tillering and enhancing seed-setting rates, thereby boosting rice production. The exogenous addition of the R3 strain primarily enhances the nitrogen-fixing capacity and sustainability of the microbial community in rhizosphere soil by elevating the α-diversity and increasing the relative abundance of key nitrogen-fixing groups (*Ralstonia*, *Azotobacter*, *Geobacter*, *Streptomyces*, and *Pseudomonas*), and that this enhancement elevates the content of available nitrogen in the rhizosphere soil. Meanwhile, inoculation with the R3 strain upregulates the expression of the genes *OsNRT1* and *OsPTR9* in rice roots, enhancing the absorption and transport of available nitrogen from the soil and thus contributing to increased rice yield. Our research elucidates the potential microbial mechanisms by which nitrogen-fixing microbial communities enhance rice production and establishes a solid theoretical foundation for future applications in sustainable agricultural production.

## Supplementary Information

Supplementary Material 1.

## Data Availability

The raw data for sequencing were deposited in the National Center for Biotechnology Information (NCBI) Sequence Read Archive (SRA) database under the project ID PRJNA1126613.
