# Supplementary material for: Mechanism of microbial action of the inoculated nitrogen-fixing bacterium for growth promotion and yield enhancement in rice (Oryza sativa L.)
Source: Adv Biotechnol (Singap). 2024 Sep 19;2(4):32. doi: 10.1007/s44307-024-00038-4 (PMC11709144; doi:10.1007/s44307-024-00038-4)
Supplement: Supplementary file 1 — Supplementary Material 1. [file 44307_2024_38_MOESM1_ESM.docx]

**Mechanism of microbial action of the inoculated nitrogen-fixing bacterium for growth promotion and yield enhancement in rice (*Oryza sativa* L.)**

Peng Li^1^, Yunhe Tian^2^, KunYang^3^, Meijie Tian^2^, Yi Zhu^4^, Xinyu Chen^2^, Ruiwen Hu^2^, Tian Qin^2^, Yongjun Liu^3^, Shuguang Peng^3^, Zhenxie Yi^2^, Zhixuan Liu^5^*, Hejun Ao^2^*, Juan Li^2^*

^1^ Hunan Soil and Fertilizer Institute, Hunan Academy of Agricultural Sciences, Changsha, 410125, China

^2^ College of Agronomy, Hunan Agricultural University, Changsha, 410128, China

^3^ Hunan Tobacco Science Institute, Changsha, 410004, China

^4^ Hunan Tobacco Company Changde Branch, Changde, 415000, China

^5^ Hunan Rice Research Institute, Hunan Academy of Agricultural Sciences, Changsha, 410125, China

* Correspondence: Zhixuan Liu ([zhixuanliu@hunaas.cn](mailto:zhixuanliu@hunaas.cn)); Hejun Ao ([aohejun@126.com](mailto:aohejun@126.com)); Juan Li ([adalee619@163.com](mailto:adalee619@163.com)).

**SM: table**

**Table S1** Physiological functions of endophytic Azotobacter R3

| Strain | Siderophore production capacity | Solubilizing phosphate ability | Nitrogen fixation function | IAA production function |
| --- | --- | --- | --- | --- |
| R3 | 1.20 | 1.20 | √ | √ |

Note: The strength of the iron-carrier/phosphorus-solubilizing capacity was expressed using the D/d value, where D is the diameter of the iron-carrier orange-yellow circle/phosphorus-solubilizing circle, and d is the diameter of the colony. Larger D/d values indicate greater iron carrier/dissolved phosphorus production capacity.

**Table S2** Nutrient content of the tested soil

| Treatment | pH | TN  /g·kg^-1^ | TP  /g·kg^-1^ | TK  /g·kg^-1^ | NH_4_-N  /mg·kg^-1^ | NO_3_-N  /mg·kg^-1^ | AP  /mg·kg^-1^ | AK  /mg·kg^-1^ | SOM  /g·kg^-1^ |
| --- | --- | --- | --- | --- | --- | --- | --- | --- | --- |
| Original soil | 6.79 | 2.44 | 2.40 | 5.18 | 38.57 | 8.90 | 15.46 | 97.15 | 28.25 |

**Table S3** Primer design

| Gene name | Primer sequence (5’-3’) | Tm(℃) | Amplicon length (bp) |
| --- | --- | --- | --- |
| eEF-2 | F:TTTCACTCTTGGTGTGAAGCAGAT  R:GACTTCCTTCACGATTTCATCGTAA | 62  62 | 103 |
| OsNRT1 | F:CGTGTCATCCCCACTCTACA  R:ACTGAGAAGACAGAACCGAGAGA | 58  60 | 176 |
| OsPTR9 | F:TCCATGAAGGGAGTTCAGAGG  R:GGGCAGCATCCTGACGAGAA | 58  62 | 174 |

**Table S4** Dry matter weight of rice plants at maturity

| Treatment | Root/g·plant^-1^ | Stem/g·plant^-1^ | Leaf/g·plant^-1^ | Grain/g·plant^-1^ |
| --- | --- | --- | --- | --- |
| CK | 7.68±0.24b | 34.88±1.06b | 11.51±0.67b | 35.34±1.17b |
| R3 | 8.73±0.16a | 37.18±1.00a | 13.40±0.91a | 38.34±0.95a |

Mean values (± S.D., n = 10) with different letters indicating significant differences between CK and R3 treatments based on Duncan test (*P* < 0.05), the same below.

**Table S5** Dissimilarity test of communities based on three non-parametric tests

| Sample | Treatment | MRPP | | ANOSIM | | PERMANOVA | |
| --- | --- | --- | --- | --- | --- | --- | --- |
|  |  | Delta | *P* | R value | *P* | F value | *P* |
| Rhizosphere | CK vs. R3 | 0.1690 | 0.001 | 0.5936 | 0.001 | 10.2859 | 0.001 |
| Endophyte | CK vs. R3 | 0.3895 | 0.016 | 0.1125 | 0.013 | 2.6757 | 0.007 |

MRPP: multi-response permutation procedure; ANOSIM: analysis of similarities; PERMANOVA: permutational multivariate analysis of variance.

**Table S6** Topological properties of the networks

| Network Indices | Soil_CK | Soil_R3 | Plant_CK | Plant_R3 |
| --- | --- | --- | --- | --- |
| Similarity threshold | 0.770 | 0.770 | 0.850 | 0.850 |
| Total nodes | 105 | 154 | 35 | 54 |
| Total links | 138 | 289 | 157 | 298 |
| R square of power-law | 0.859 | 0.815 | 0.073 | 0.14 |
| Average degree (avgK) | 2.629 | 3.753 | 8.971 | 11.037 |
| Average clustering coefficient (avgCC) | 0.13 | 0.13 | 0.249 | 0.197 |
| Average path distance (GD) | 4.572 | 4.411 | 2.237 | 2.121 |
| Geodesic efficiency (E) | 0.275 | 0.279 | 0.567 | 0.553 |
| Harmonic geodesic distance (HD) | 3.641 | 3.589 | 1.764 | 1.809 |
| Maximal degree | 9 | 15 | 17 | 28 |
| Centralization of degree (CD) | 0.062 | 0.074 | 0.25 | 0.332 |
| Maximal betweenness | 988.92 | 1486.826 | 79.92 | 328.987 |
| Centralization of betweenness (CB) | 0.164 | 0.111 | 0.108 | 0.221 |
| Maximal stress centrality | 3094 | 9101 | 1094 | 1909 |
| Centralization of stress centrality (CS) | 0.523 | 0.708 | 1.548 | 1.18 |
| Maximal eigenvector centrality | 0.366 | 0.316 | 0.278 | 0.269 |
| Centralization of eigenvector centrality (CE) | 0.315 | 0.272 | 0.135 | 0.158 |
| Density (D) | 0.025 | 0.025 | 0.264 | 0.208 |
| Reciprocity | 1 | 1 | 1 | 1 |
| Transitivity (Trans) | 0.097 | 0.081 | 0.314 | 0.275 |
| Connectedness (Con) | 0.629 | 0.78 | 1 | 1 |
| Efficiency | 0.974 | 0.976 | 0.758 | 0.807 |
| Lubness | 1 | 1 | 1 | 1 |

**Table S7** Summary of the basic taxonomies of putative keystone species in the networks

| Network category | Treatment | OUT_ID | Classification | |
| --- | --- | --- | --- | --- |
|  |  |  | Phylum | Genus |
| Module hubs | CK_Soil | OTU_113 | Actinobacteria | Unclassified |
|  | R3_Soil | OTU_10 | Actinobacteria | Unclassified |
| Network hubs | R3_Soil | OTU_211 | Firmicutes | Unclassified |
|  | R3_Root | OTU_29 | Proteobacteria | Unclassified |
| Connectors | CK_Soil | OTU_193 | Actinobacteria | Corynebacterium |
|  | R3_Soil | OTU_88 | Proteobacteria | Unclassified |
|  |  | OTU_114 | Proteobacteria | Unclassified |
|  |  | OTU_125 | Proteobacteria | Acidithiobacillus |
|  |  | OTU_140 | Proteobacteria | Myxococcus |
|  |  | OTU_149 | Firmicutes | Unclassified |
|  |  | OTU_162 | Proteobacteria | Chromobacterium |
|  |  | OTU_166 | Planctomycetes | Urbifossiella |
|  |  | OTU_185 | Cyanobacteria | Zehria |
|  |  | OTU_215 | Actinobacteria | Unclassified |
|  | CK_Root | OTU_55 | Proteobacteria | Unclassified |
|  |  | OTU_57 | Proteobacteria | Moraxella |
|  |  | OTU_63 | Actinobacteria | Streptomyces |
|  |  | OTU_75 | Proteobacteria | Sinorhizobium |
|  | R3_Root | OTU_38 | Proteobacteria | Azospirillum |
|  |  | OTU_66 | Proteobacteria | Pseudomonas |
|  |  | OTU_82 | Proteobacteria | Escherichia |

**Table S8** Grouping and its factor composition in the path model

| Treatment | Factor |
| --- | --- |
| α diversity of rhizosphere diazotrophs | Shannon, Simpson, Richness, Pielou_evenness |
| α diversity of endosphere diazotrophs | Shannon, Simpson, Richness, Pielou_evenness |
| Keystone taxa of rhizosphere | p_Actinobacteria, p_Cyanobacteria, g_Ralstonia, g_Azotobacter, g_Geobacter, g_Pseudomonas, g_Streptomyces |
| Keystone taxa of endosphere | p_Actinobacteria, p_Cyanobacteria, g_Frankia |
| Genes related to nitrogen absorption and transport | OsNRT1, OsPTR9 |
| Available nitrogen content in soil | NO_3_^-^-N, NH_4_^+^-N |
| Theoretical yield of rice | Theoretical yield of rice |

Note: This grouping was used for the Partial Least Squares Path Modeling (PLS-PM) analysis in this study.

**SM: Figure**

**
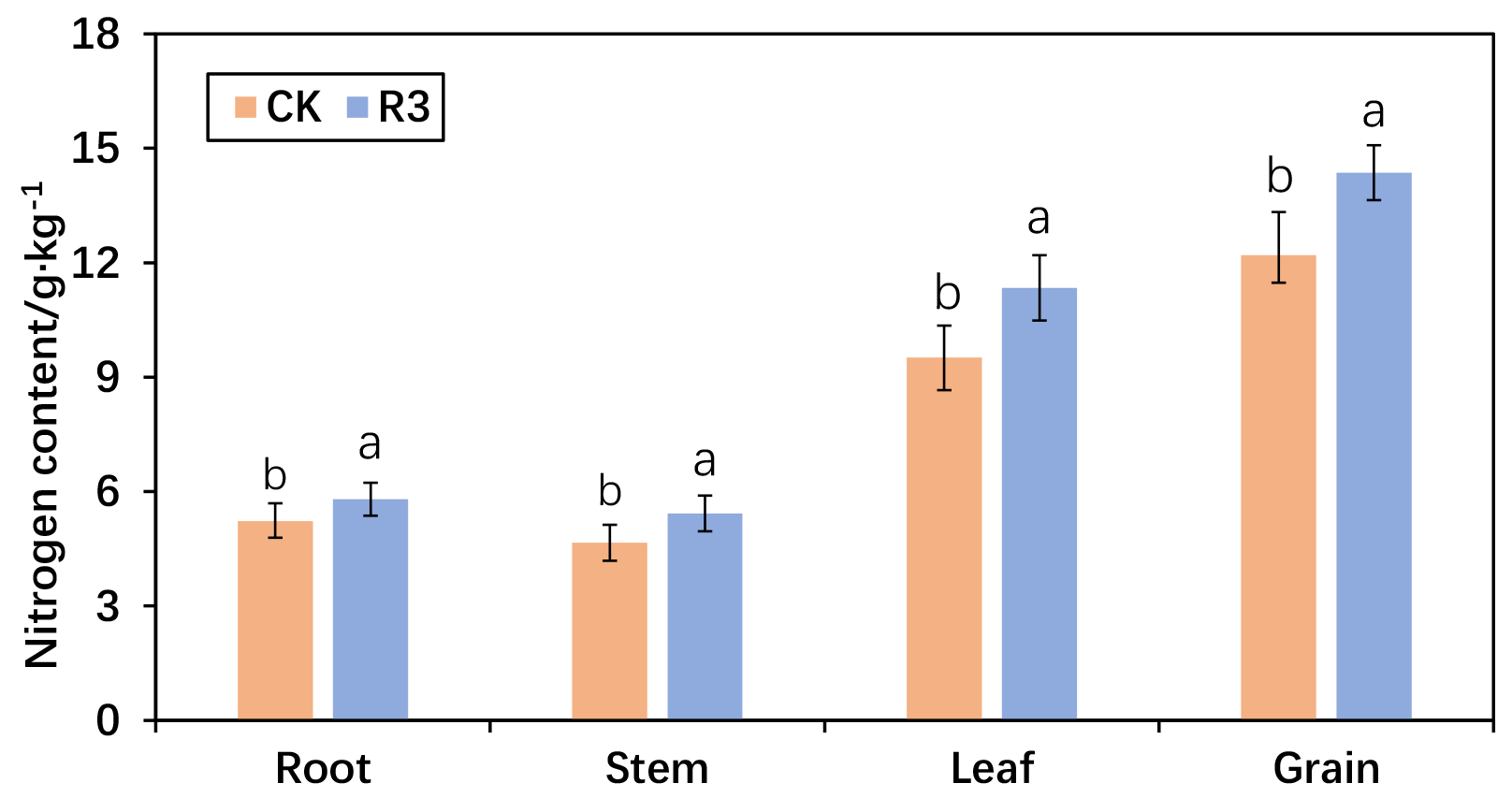
**

**Fig. S1** Nitrogen content in various parts of rice plants. Different letters indicate that N content was significantly different between treatments (*P* < 0.05).

**
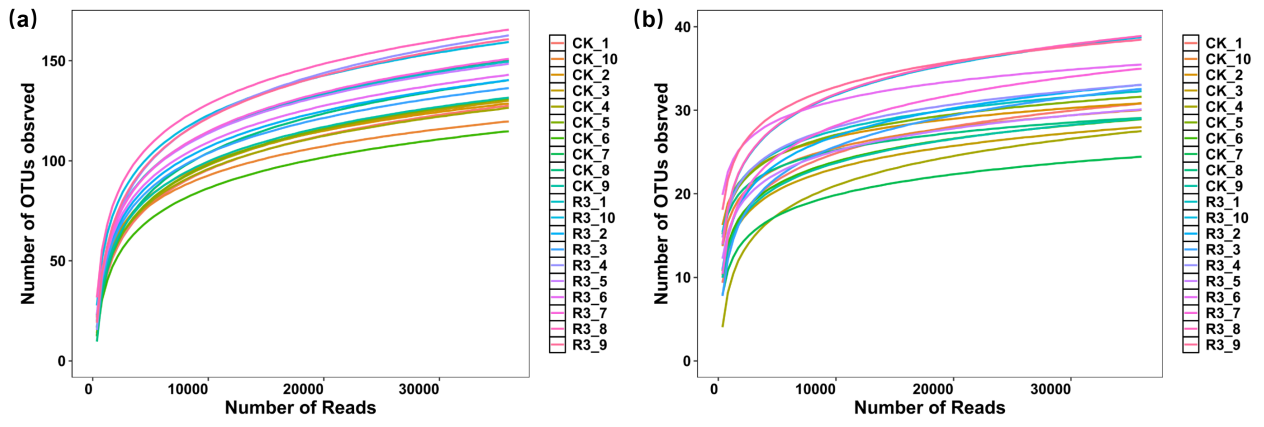
**

**Fig. S2** Rarefaction curves of observed number of OTUs against the number of *nifH* gene sequences retrieved from the rice rhizosphere (a) and root endosphere (b).


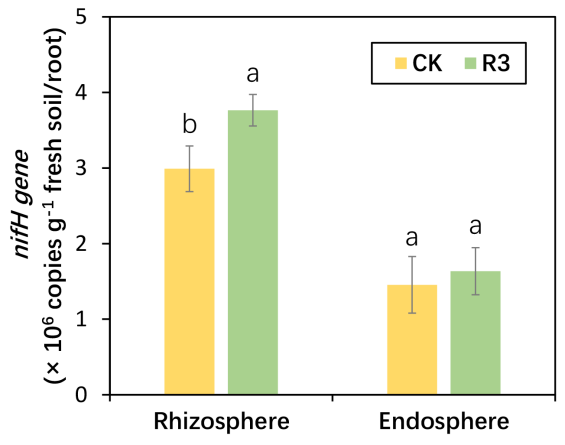


**Fig. S3** nifH gene abundance in rhizosphere and root endosphere of rice. Different letters indicate that abundance was significantly different between treatments (*P* < 0.05).

**
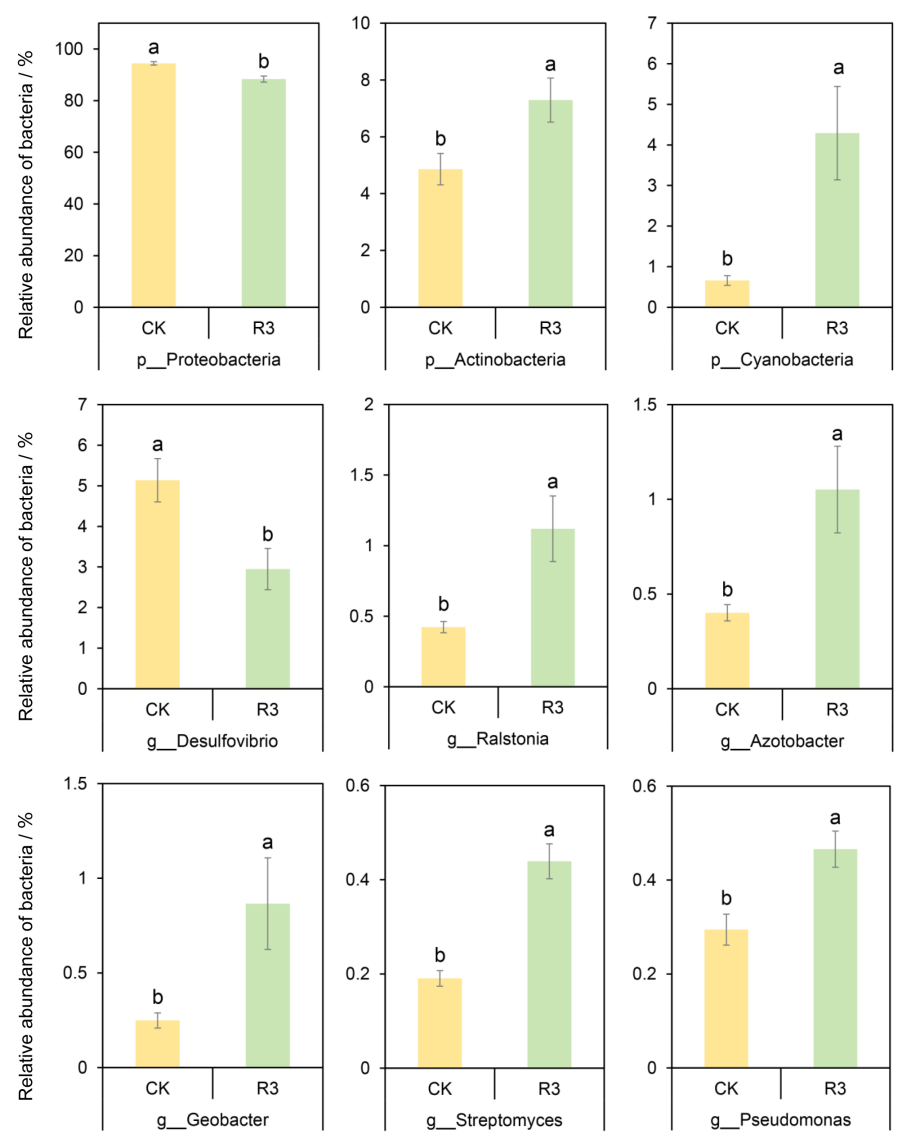
**

**Fig. S4** The relative abundance of nitrogen-fixing bacteria (phylum and genus) in rice rhizosphere soil. Different letters indicate that the relative abundance of the taxon is significantly different between treatments (*P* < 0.05).

**
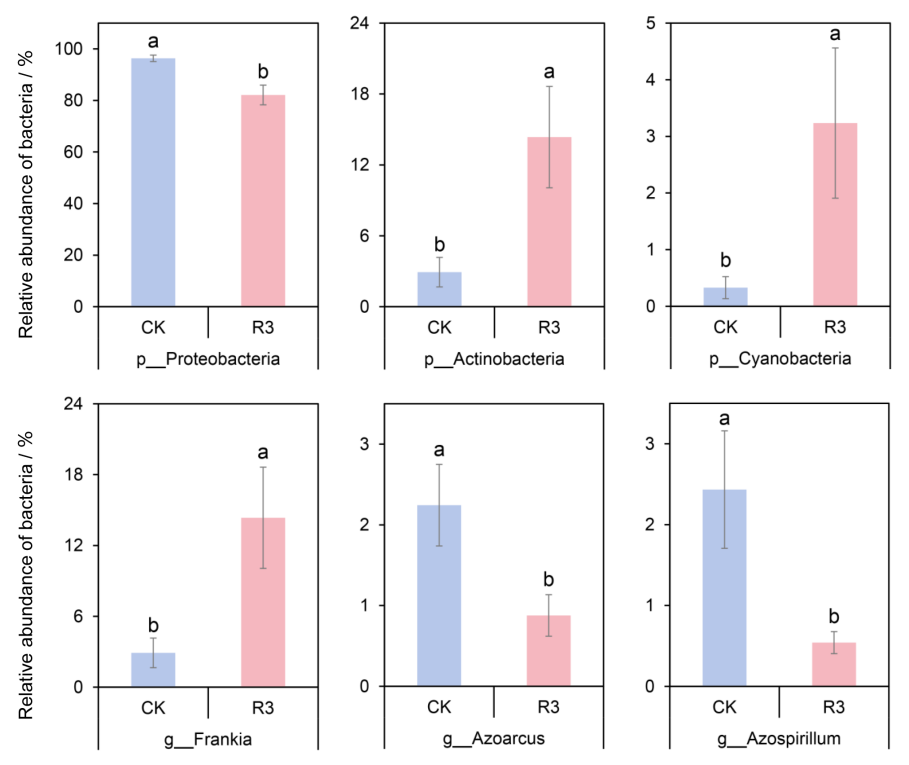
**

**Fig. S5** The relative abundance of nitrogen-fixing bacteria (phylum and genus) in rice roots. Different letters indicate that the relative abundance of the taxon is significantly different between treatments (*P* < 0.05).
